# In Press—A New and Concise Text-Book on Hygiene

**Published:** 1894-09

**Authors:** 


IN PRESS—A NEW AND CONCISE TEXT-BOOK ON HYGIENE—By Mary Taylor Bissell, Professor of Hygiene in the Woman's Medical College of the New York Infirmary. 8vo, Cloth, $2.00.

The consideration of the subject is based on the modern scientific theory of the origin of disease and the methods of prevention deduced from this. It is intended to be practical and to accord with the best American usage. It is designed to supply the want of a concise text-book, both for medical students and for use in advanced schools and colleges.

Its treatment includes the subjects of Air, Water, Soil, Foods, The Hygiene of the Dwelling, Personal Hygiene and Preventable Diseases.

Specimen pages will be sent postpaid to teachers.

Special terms for examination and introduction.

8vo, Cloth, price $2.00.

The Baker & Taylor Co., Publishers, 5 & 7 East Sixteenth street, New York.

---

A USEFUL FORMULA.—Dr. R. T. Colliver, Greenup, Ill., prescribes the following formula in cases of hemorrhoids, pruritus ani, ulceration of the uterus, leucorrhea, and excessive secretion of the corona glandis:

℞ Ext. Pinus Canadensis (Kennedy's)........................1 ℥
Glycerini........................................1 ℥
Aquæ dest........................................1 ℥
M. Sig.—Apply locally.

